# Post cholecystectomy syndrome: Role of cystic duct stump and re-intervention by laparoscopic surgery

**DOI:** 10.4103/0972-9941.43090

**Published:** 2008

**Authors:** Om Tantia, Mayank Jain, Shashi Khanna, Bimalendu Sen

**Affiliations:** Department of Minimal Access Surgery, ILS Multispeciality Clinic, Kolkata- 700 064, West Bengal, India

**Keywords:** Cystic duct stump calculi, stump cholecystitis, stump syndrome

## Abstract

Laparoscopic cholecystectomy is the most common surgery performed for symptomatic gallstones. However even after surgery, symptoms may persist in some patients. Various causes for such post-cholecystectomy syndrome have been noted. We report our experience of seven such patients with post-cholecystectomy syndrome where on investigations, presence of stone in the biliary tree could be confirmed along with remnant gall-bladder. All these patients underwent completion cholecystectomy with removal of the stones by laparoscopic surgery and had good post-operative result. The patients were followed-up from three months to one year and all were asymptomatic till their last follow-up.

## INTRODUCTION

Laparoscopic cholecystectomy is the most common surgery performed for symptomatic gallstones. However, symptoms may persist even after surgery in some patients. Various causes for such symptoms have been identified in the literature and are grouped together as post cholecystectomy syndrome.

We report our experience of post cholecystectomy syndrome in seven patients of which five had calculi in the remnant gall bladder (GB) and two had remnant gall bladder with common bile duct (CBD) stone. All cases were managed laparoscopically by completion cholecystectomy along with laparoscopic CBD exploration for two cases with CBD stone.

## MATERIALS AND METHODS

Data was retrospectively searched from January 2001 to December 2006 for post-cholecystectomy cases, which underwent laparoscopic completion cholecystectomy and seven such cases were found (two male and five female). All had undergone previous cholecystectomy varying from one year to 40 years back. Of these, six were open cholecystectomy and one was a laparoscopic cholecystectomy (LC). Of the seven patients, six were operated at other centres and were referred or directly came with recurrent symptoms. Two of these were referred with CBD calculus with gall bladder stump, two with cystic duct stump calculi two with gall bladder stump calculi as per their ultrasound reports. One patient was operated in the same institute and partial LC was done one year back in lieu of Mirizzi Syndrome. This patient was also diagnosed to have GB stump calculi. Details of previous surgery notes (per-operative findings) could not be obtained in the cases operated at other centres.

All patients were symptomatic for more than 3 months prior to revision cholecystectomy.

Technique - initial access was by close technique - trans umbilical in five cases and through palmer's point in two cases. Optical port in these two cases was made at umbilicus after clearing adhesions. Other ports were made strictly under vision and the port positions were as in standard LC.

- 10 mm Epigastric - Right hand working port
- 5 mm Right mid clavicular line just below the costal margin - Left hand working port
- 5 / 10 mm port anterior axillary line at the level of umbilicus - For liver retraction.

Dissection was started laterally from the inferior margin of liver and progressively moving medially. After separating the colon and omentum from liver with blunt and sharp dissection and the use of HARMONIC SCALPEL (Ethicon Endosurgery, Cincinnati, Ohio, USA), remnant gall bladder was dissected. The remnant GB was excised and the cystic duct was ligated with 3-0 VICRYL (Ethicon Endosurgery, Cincinnati, Ohio, USA). In all seven patients cystic artery could be dissected and clipped separately.

## RESULTS

The demographic data, symptoms, investigations (biochemical and radiological), therapeutic procedures and operative time of the seven patients who underwent completion cholecystectomy is mentioned in Table 1.

**Table 1:** Demographic data, symptoms, investigations (biochemical & radiological), therapeutic procedure and operative time

S. No.	Age	Sex	Symptoms	Past surgery	Biochemical (Bil, SGOT/SGPT, ALP)	Radiological		Therapeutic procedure	Operative time (mins)
								
						USG	ERC / MRCP		
1	50	F	Pain right upper abdomen - 1 yr	OC - 7 yrs back	0.4*(0.2**), 18/30, 114	Dilated cystic duct stump with stone CBD - 6mm	-	Completion cholecystectomy	55
2	65	M	Pain epigastrium + nausea - 2 yrs	OC - 10 yrs back	0.6*(0.2**), 24/32, 214	Stone in gall bladder stump. CBD - 6mm	-	Completion cholecystectomy	40
3	40	F	Pain epigastrium - 6 months. Fever and vomiting - 15 days	OC - 2 yrs back	2.0*(1.6**) 30/26, 1196	Gall bladder stump without any calculus Dilated CBD (12mm) with stone.	ERC - Large CBD stone which could not be extracted. Wide sphicterotomy & biliary stenting was done.	ERC - Faiure - completion cholecystectomy + Lap CBDE	100
4	64	F	Pain upper abdomen - 2 yrs	OC - 20 yrs back	2.1*(1.6**) 26/13, 207	Calculi in cystic duct stump. CBD - 6mm.	MRCP - Residual cystic duct stump with calculi	Completion cholecystectomy	40
5	22	F	Pain right upper abdomen - 3 months	LC - 1 yrs back	0.7*(0.4**) 24/38, 220	Calculi in gall bladder stump with ? CBD stone. CBD - 10mm	-	Completion cholecystectomy + POC (biliary passage clear dye passed smoothly to the duodenum)	50
6	70	M	Pain right upper abdomen + nausea - 2 yrs	OC - 40 yrs back	1.3*(1.1**) 44/51, 5011	Gall bladder stump without any calculi. CBD - 14mm. Multiple CBD stones	ERC - Sphicterotomy was done but stones could not be extracted	ERC - Faiure - completion cholecystectomy + Lap CBDE + Choledocho-uodenostomy	110
7	57	F	Chronic pain upper abdomen - 3 months	OC - 10 yrs back	1.8*(1.4**) 40/36, 470	Gall bladder stump without any calculus CBD - (6mm)	MRCP - Dilated fluid filled structure in gall bladder fossa with intra-luminal defect. ? Dilated cystic duct with intra-luminal calculus	Completion cholecystectomy	40

ALP - Alkaline Phosphate, USG - Ultrasound sonography, ERC - Endoscopic Retrograde Cholangiogram, MRCP - Magnetic Resonance Cholangio Pancreatogram, OC - Open Cholecystectomy, LC - Laparoscopic Cholecystectomy, POC - Per-operative Cholangiogram, CBD - Common Bile Duct, Bil - serum bilirubin

* Serum bilirubin (Total)

** Serum bilirubin (Conjugated)

**Figure 1:**
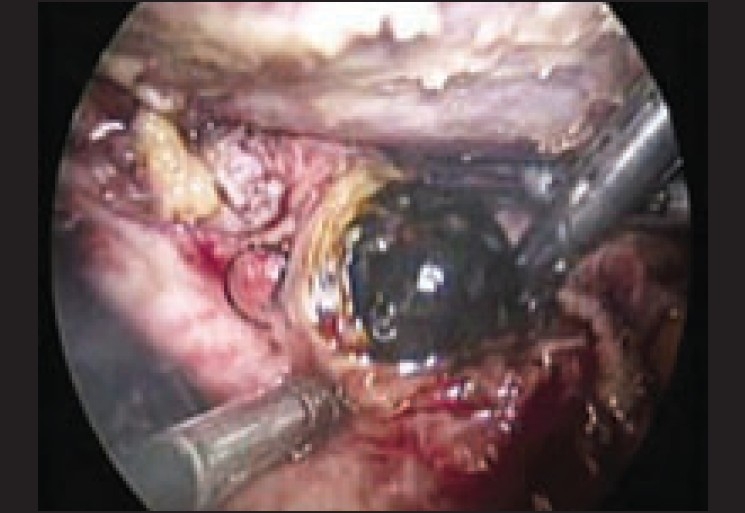
CBD stone in a patient with cystic duct stump undergoing laparoscopic CBD exploration

**Figure 2:**
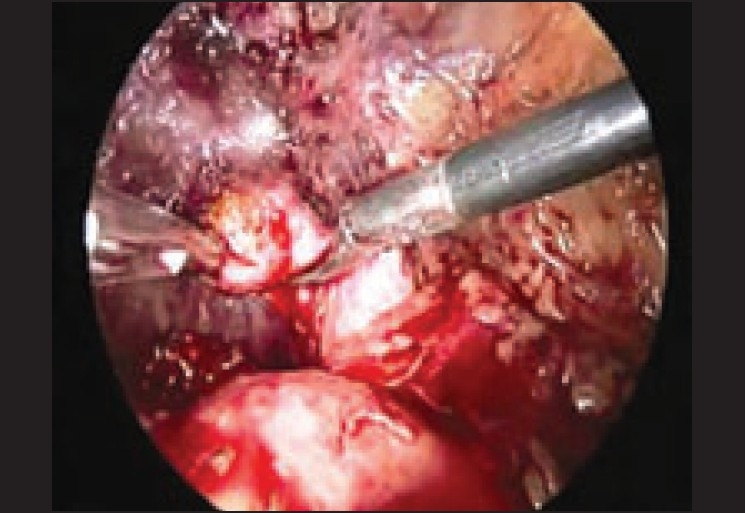
Stump cholecystectomy in a patient with a cystic duct stump with stone

Two patients had direct radiological evidence of CBD calculi along with a large stump without any calculus. Endoscopic retrograde cholangiogram (ERC) and stone extraction was attempted in both these patients but failed due to a large calculus in CBD in one and multiple stones in the other. These patients underwent laparoscopic completion cholecystectomy along with laparoscopic common bile duct exploration (LCBDE) [Figure 1]. In the first case, CBD clearance was confirmed with intra-operative choledochoscopy and primary closure of the CBD was done with antegrade stenting. In the second case, after clearing the CBD laparoscopic choledochoduodenostomy was done due to its large stone load. Two patients with stump calculi had suspicion of CBD calculi on the basis of their biochemical investigations but had normal CBD diameter and therefore underwent pre-operative magnetic resonance cholangio-pancreatography (MRCP) which depicted clear CBD without any narrowing. Both these patients then underwent laparoscopic completion cholecystectomy. In two patients there was no evidence of CBD stone either on biochemical or radiological investigation and dilated stump with calculi was the only abnormality, therefore laparoscopic completion cholecystectomy was done [Figure 2]. One patient had dilated CBD (10 mm) on USG but there was no biochemical / radiological evidence of calculi and so was planned for laparoscopic completion cholecystectomy with per-operative cholangiography (POC) to rule out any stone in the major bile duct which if present could be managed by LCBDE. Since no stone was found on POC, only completion cholecystectomy was done.

The mean duration of surgery was 62 min. The outcome of all the patients was good. There was no mortality and the mean post-operative stay was 3.1 days.

### Follow-up

The patients were followed-up on day 7, 30, 90 and at one year. All seven patients followed on Day 90 but only five followed at one year.

All patients were asymptomatic at their last follow-up.

## DISCUSSION

Laparoscopic cholecystectomy is an established operation for symptomatic gall stone disease. It provides total relief of pre-surgical symptoms in up to 85% of patients.[1] However about 5% of patients may experience severe episodes of upper abdominal pain similar to those that they had prior to cholecystectomy.[1] These symptoms may be due to biliary stricture, retained / recurrent biliary calculi, stenosis or dyskinesia of sphincter of Oddi, cystic neuroma, remnant gall bladder / cystic duct stump calculi etc. and are together grouped as post cholecystectomy syndrome.

Cystic duct remnant defined as residual duct greater than 1 cm in length may in presence of stones cause post-cholecystectomy syndrome.[2] The role of cystic duct stump in post cholecystectomy syndrome was evaluated by Rogy *et al*[3] in 322 patients undergoing second operation on bile duct after cholecystectomy and found 35 (10.8%) patients with long cystic duct stump (>1.5 cm). Of those, 24 had other pathological findings besides long stump. Of the remaining 11, eight had stone in partial gall bladder or cystic duct stump, one had suture granuloma, one had fistula between cystic duct and duodenum and only one patient had long cystic duct as the sole pathological finding. They concluded that cystic duct stump is hardly ever a cause of recurrent symptoms in itself and total excision of cystic duct does not eliminate the existence of post cholecystectomy symptoms. Walsh *et al*[4] reviewed seven cases with calculi retained in gall bladder and cystic duct remnants that presented with recurrent biliary symptoms. They concluded that retained gall bladder and cystic duct calculi can be a source of recurrent biliary pain and said that this entity can be prevented by accurate identification of gall bladder cystic duct junction at cholecystectomy. Although rare, recurrent cholelithiasis involving cystic duct stump may cause massive dilatation and should be a differential diagnosis of post-cholecystectomy syndrome.[5]

In our series of seven patients operated for post cholecystectomy syndrome, all had an evidence of calculi in the biliary tree. Five patients had GB stump (three with calculi and two without calculi) and two had cystic duct stump with calculi on ultrasound but all were found to have remnant GB intra-operatively. This difference in nomenclature by radiologist may be taken as difference in opinion on what they see at GB fossa in a previously operated case and can only be confirmed per-operatively. Since both the cases with cystic duct stump calculi on ultrasound were diagnosed as remnant GB with calculi intraoperatively, we also believe that the long stump alone may not be the cause of recurrent symptom and the possibility of finding a remnant GB must be kept in mind with such pre-operative diagnosis. The mean duration between previous surgery and revision surgery was approximately 13 years and the mean duration between onset of symptoms and revision surgery was 14 months. This shows that the patient may present late after the initial surgery and when symptomatic, the diagnosis may be over-looked resulting in delayed treatment.

In this era of laparoscopic surgery, where surgery favours a long cystic duct remnant, one should be aware of cystic duct stones as a possible cause of post-cholecystectomy syndrome.[2] MRCP emerges as the optimal method for evaluating the biliary tree in these cases as it is a non-invasive method.[2]

After incomplete cholecystectomy the cystic duct stump and Calot's triangle is usually embedded in inflamed scar tissue.[1] So it was thought that the surgical risk is too high with laparoscopic technique to re-operate on these cases.[1] However with modern instruments and advances in laparoscopic surgery and increasing experience of surgeons, even these can be operated laparoscopically. It has now been suggested that it is safe and feasible to remove the gall bladder or gall bladder remnants in such patients laparoscopically.[6] Clemente *et al* also described the feasibility of laparoscopic removal Gall Bladder remnant and cystic duct stump.[7]

Chowbey *et al* recently reported five patients who underwent laparoscopic re-intervention after previous surgery for gall stone disease under them. Their mean operative time was 42 min. They believe re-intervention may be required for patients with residual gallstones whose symptoms recur after gall bladder surgery.[6]

In our series all cases were treated laparoscopically without conversion with a mean operative time of 62 min by completion cholecystectomy with complementary procedure if needed (LCBDE or POC) as we also believe that despite adhesions in the gallbladder fossa, these patients can managed well with laparoscopic surgery.

## CONCLUSION

We would like to emphasise the importance of proper dissection and identification of gallbladder - cystic duct junction to completely remove the GB and prevent recurrent symptoms. Cystic duct stump calculi diagnosed on ultrasound as a cause of these symptoms may actually be in the remnant gall bladder. Further, patients with recurrent symptoms and proven stones should be re-operated and laparoscopic surgery is no more a contra-indication for these revision surgeries.

## References

[1] Rozsos I, Magyarodi Z, Orban P (1997). Cystic Duct Syndrome and minimally invasive surgery. Orv Hetil.

[2] Shaw C, O'Hanlon DM, Fenlon HM, McEntee GP (2004). Cystic duct remnant and the post-cholecystectomy syndrome. Hepatogasroenterology.

[3] Rogy MA, Fugger R, Herbst F, Schulz F (1991). Reoperation after cholecystectomy: The role of the cystic duct stump. HPB Surg.

[4] Walsh RM, Ponsky JL, Dumot J (2002). Retained gall bladder/cystic duct remnant calculi as a cause of post cholecystectomy pain. Surg Endosc.

[5] Mergener K, Clavien PA, Branch MS, Baillie J (1999). A Stone in a grossly dilated cystic Duct Stump: A rare cause of post cholecystectomy pain. Am J Gastroenterol.

[6] Chowbey PK, Bandyopadhyay SK, Sharma A, Khullar R, Soni V, Baijal M (2003). Laparoscopic reintervention for residual gallstone disease. Surg Laparosc Endosc Percutan Tech.

[7] Clemente G, Giuliante F, Cadedu F, Nuzzo G (2001). Laparoscopic removal of Gall Bladder remnant and long cystic Stump. Endoscopy.

